# Modification of Lightweight Aggregates’ Microstructure by Used Motor Oil Addition

**DOI:** 10.3390/ma9100845

**Published:** 2016-10-18

**Authors:** Małgorzata Franus, Grzegorz Jozefaciuk, Lidia Bandura, Krzysztof Lamorski, Mieczysław Hajnos, Wojciech Franus

**Affiliations:** 1 Department of Geotechnical Engineering, Faculty of Civil Engineering and Architecture, Lublin University of Technology, Nadbystrzycka 40, 20-618 Lublin, Poland; m.franus@pollub.pl; 2 Department of Physical Chemistry of Porous Materials, Institute of Agrophysics, Polish Academy of Sciences, Doświadczalna 4, 20-290 Lublin, Poland; g.jozefaciuk@ipan.lublin.pl (G.J.); k.lamorski@ipan.lublin.pl (K.L.); m.hajnos@ipan.lublin.pl (M.H.)

**Keywords:** lightweight aggregates, used oil, pore structure, microtomography, porosimetry

## Abstract

An admixture of lightweight aggregate substrates (beidellitic clay containing 10 wt % of natural clinoptilolite or Na-P1 zeolite) with used motor oil (1 wt %–8 wt %) caused marked changes in the aggregates’ microstructure, measured by a combination of mercury porosimetry (MIP), microtomography (MT), and scanning electron microscopy. Maximum porosity was produced at low (1%–2%) oil concentrations and it dropped at higher concentrations, opposite to the aggregates’ bulk density. Average pore radii, measured by MIP, decreased with an increasing oil concentration, whereas larger (MT) pore sizes tended to increase. Fractal dimension, derived from MIP data, changed similarly to the MIP pore radius, while that derived from MT remained unaltered. Solid phase density, measured by helium pycnometry, initially dropped slightly and then increased with the amount of oil added, which was most probably connected to changes in the formation of extremely small closed pores that were not available for He atoms.

## 1. Introduction

Lightweight aggregates (LWAs) are grained materials, produced from different minerals (ordinary soil clay, perlite, vermiculite, natural and synthetic zeolites, and others) by rapid sintering/heating at high temperatures, up to 1300 °C. To achieve an appropriately expanded material, two conditions are necessary: the presence of substances that release gases at high temperature; and a plastic phase with the adequate viscosity, able to trap the evolved gases [1]. The expanded clay aggregates are nonflammable and highly resistant against chemical, biological and weather conditions. Their highly porous structure is represented mainly by closed pores surrounded by glassy coatings formed at the thermal transformation of clay minerals. As a consequence, LWAs have a small density, low thermal conductivity and sound dampening characteristics [2,3,4,5,6]; they thus have a broad range of applications in the construction and concrete industry, geotechnics, gardening or agriculture [4,7,8,9,10,11,12,13].

Much effort has been recently invested to modify the LWAs’ structure using a variety of materials including different fly ashes, glass, sewage or industrial sludges, mining residues, polishing residues, used sorbents and/or contaminated soils [3,4,13,14,15,16,17,18,19,20,21,22,23,24,25,26,27]. Some of these materials may contribute to the foaming or bloating of LWAs during sintering, thus increasing their porosity [28].

In our previous papers, we found that zeolite minerals, among a broad range of industrial applications [29], are very efficient sorbents for oil land spills and BTX (benzene, toluene and xylenes) removal [30,31,32], however significant amounts of waste materials are simultaneously produced. In preliminary experiments, we applied used zeolitic sorbents containing motor oil for LWAs’ production, finding that they highly modify the LWAs’ structure, however, a question arose: what was the input of the oil itself to the structure formation? Therefore, at present, we attempted to evaluate this effect using different admixtures of used motor oil to the LWAs’ substrates.

## 2. Materials and Methods

### 2.1. Substrates

Beidellitic clay deposits taken from Budy Mszczonowskie, Poland and two zeolitic sorbents-natural clinoptilolite, coming from Sokyrnytsia, Ukraine, and synthetic Na-P1 were used. Zeolite Na-P1 was synthetized from coal fly ash, the chemical composition of which was suitable for the hydrothermal conversion process [33]. The synthesis of Na-P1 was performed according to Wdowin et al. [34]. The clay deposit contained around 50% beidellite, 25% quartz, 9%, 7% illite, 7% feldspars and less than 2% iron hydroxides. Clinoptilolitic rock contained about 75% of pure clinoptilolite phase, slight amounts of Opal-CT, quartz, potassium feldspars, and mica [35]. Na-P1 contained around 80% of pure zeolite phase [36], residual amount of quartz, mullite and remainders of unreacted amorphic species. Used motor oil, Total Rubia Tir 6400 15W-40, designed for diesel engines was used as the additive. The oil was taken from truck service BIOMIX (Lublin, Poland).

### 2.2. Lightweight Aggregates’ Preparation

Gently ground clay deposit, 0.5 mm sieved and dried at 105 °C, was used in its original state and as mixtures containing 10% of each of the zeolite (addition of more than 15% of the zeolites drastically reduced the mechanical strength of the produced aggregates). To the above mineral matrices, used motor oil was added to reach its final concentrations of 0%, 1%, 2%, 4%, and 8% by weight. Next, each substrate was carefully homogenized and wetted with water to obtain plastic masses from which spherical granules of around 1 cm diameter were formed by hand. The granules were air-dried at room temperature for 24 h, then at 50 °C for 2 h, and finally at 105 °C for 12 h. The dry granules were placed into a laboratory furnace SM-2002 “Czylok”, subjected to sintering at 1170 °C for 30 min, left in the furnace for cooling to approximately 100 °C and stored in closed vessels. The aggregates, prepared from the natural clay deposit, will be abbreviated further as S; these admixed with the clinoptilolite as S + Clin and with Na-P1 as S + NAP.

### 2.3. Methods of Characterization

Mineralogical composition of the substrates and the obtained lightweight aggregates was examined by X-ray diffraction (XRD) analysis using a X’pert PROMPD spectrometer (Panalytical, Almelo, The Netherlands) with a PW 3050/60 goniometer (Panalytical), a Cu lamp and a graphite monochromator within a 2θ range of 5°–65°. Identification of mineral phases was based on the JCPDS-ICDD database. Bulk density of the aggregates was estimated from their volumes (measured by immersion in mercury) and masses (weighing). Solid phase densities were measured by helium pycnometry using an AccuPyc II 1340 Pycnometer, provided by Micromeritics (Norcross, GA, USA). Nitrogen adsorption isotherms were measured at liquid nitrogen temperature using an ASAP 2020MP manufactured by Micromeritics. The scanning electron microscope (SEM) images of the tested materials were taken using an FEI Quanta 250 FEG microscope (FEI, Hilsboro, OR, USA) for a one square millimeter area located in the center of the broken aggregates.

X-ray computational microtomography (MT) was applied to obtain three-dimensional (3D) scans of the studied LWAs using a Nanotom S device (General Electrics, Fairfield, CT, USA). The X-ray source with a Molybdenum target, operated at a 230 µA cathode current and 60 kV voltage was used for X-ray generation. The scanning process consisted of two stages: an initial pre-scan and a main measurement scan. Prior to the final scan measurement, each sample was subjected to a short, 40 min pre-scanning to heat it up and reach thermal stability, maintained further during the main scan which lasted 150 min. The scanned specimens were dry, so the only effect of heating by X-rays on the measurement could be caused by the thermal elongation of the sample holder. Pre-scan eliminated this problem. During the main scan, 2400 two-dimensional (2D) cross-section images were acquired with the spatial resolution (voxel size) of about 0.0063 mm and then used for three-dimensional (3D) porous space reconstruction. The Resulting 3D 16-bit gray level images represented the spatial structure of the specimens. Image analysis techniques were used for further processing. Initially the bit depth of images was reduced from 16-bit to 8-bit. After that, a 3D median filter with uniform kernel and a diameter equal to 3 pixels, was used for noise reduction. The next step was the thresholding procedure which utilized the Otsu algorithm. Thresholded images had 1-bit color depth with blacks representing pores. These pre-processing steps were done using ImageJ software (U.S. National Institutes of Health, Bethesda, MA, USA) and for further analysis Avizo software (FEI) was used. Some of the pores were connected with others. A 3D watershed based segmentation algorithm and then labelling algorithm were used to separate them into individual pores. After that, geometrical characteristics of pores were calculated: equivalent diameter (a diameter of the sphere with the same volume as a pore), volume, surface and fractal dimension of pores according to the maximal ball (MB) method [37]. Average data, calculated from three 3D images, are considered further. Microtomograpic images were also applied to the alternative estimation of bulk densities: the LWAs’ volumes were determined from MT scans and their masses by weighing.

Mercury intrusion porosimetry (MIP) tests were performed using AutoPore IV 9500 apparatus provided by Micromeritics for pressures ranging from ca. 0.1 to 200 MPa (pore radii from ca. 10.0 to 3.8 × 10^−3^ µm). The intrusion volumes were measured at stepwise increasing pressures, allowing equilibration at each pressure step. The maximum deviations between the mercury intrusion volumes were not higher than 6.9% and they occurred mainly at low pressures (largest pores). The volume of mercury *V* (mm^3^/g) intruded at a given pressure *P* (Pa) gave the pore volume that can be accessed. The intrusion pressure was translated on an equivalent pore radius *R* (m) following the Washburn equation:

*P* = −*A*σ_m_cosα_m_/*R* (1)

where σ_m_ is the mercury surface tension, α_m_ is the mercury/solid contact angle (taken as 141.3° for all studied materials) and *A* is a shape factor (equal to 2 for the assumed capillary pores).

Knowing the dependence of *V* vs. *R*, a normalized pore size distribution, χ(*R*), was calculated and expressed in the logarithmic scale [38]:

χ(*R*) = 1/*V*_max_·d*V*/dlog(*R*) (2)

Knowing χ(*R*), the average pore radius, *R*_av_, was calculated from:

*R*_av_ = ∫*R* χ(*R*) d*R* (3)

If a range of pore sizes, wherein the pore volume depends on the power of the pore radius, could be found, this was interpreted in terms of pore surface fractal scaling. In this case, the dependence of log(d*V*/d*R*) against log*R* was plotted and, from the slope of its linear part, the fractal dimension of the pore surface, *D*s was derived as [39]:

*D*s = 2 − slope (4)

To define the linear range of fractality, the procedure of Yokoya et al. [40] was applied.

The apparent solid phase skeletal densities of the samples, SSD, (that are lower than true skeletal densities due to the residence of the finest pores in the solid phase which are not filled by mercury at its highest pressure) were calculated by a porosimetric data analysis program provided by the equipment manufacturer.

## 3. Results and Discussion

Extremely low nitrogen adsorption was measured, thus the calculated surface areas of the produced LWAs were less than a few square meters per gram and evaluation of microstructural parameters was not possible. This may indicate that either the glassy phase, produced during heating, has an extremely low and flat surface or that the closed intra-aggregate pores are not available for nitrogen molecules.

**Figure 1:**
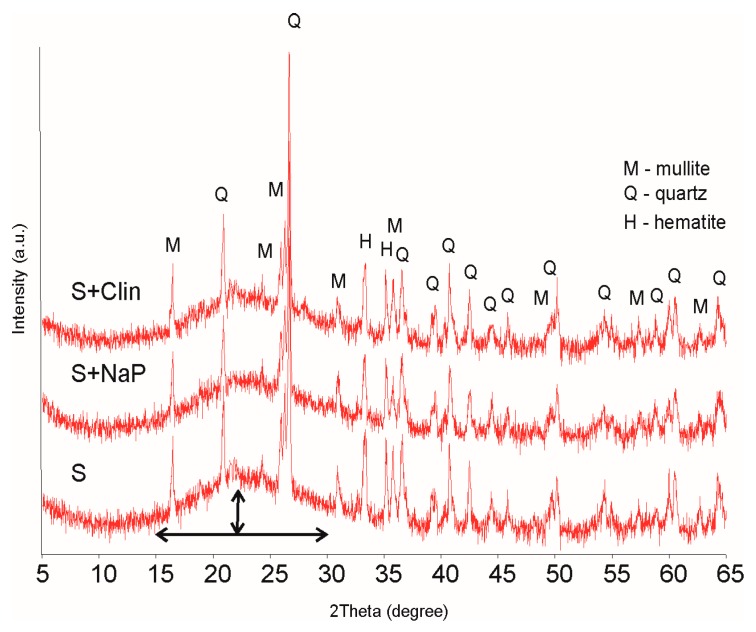
X-ray diffraction (XRD) patterns of the control aggregates.

Exemplary XRD patterns of three control LWAs: S, S + Clin, S + NAP, are presented in Figure 1. All LWAs exhibit very similar spectra regardless of the oil content and the composition of mineral substrates, showing main mineral components: mullite (*d*_hkl_ 3.39, 5.41, 3.42, and 2.21 Å) and quartz (3.34, 4.25, and 1.81 Å). The presence of mullite is an effect of the melting of the original clay minerals (beidellite, illite, kaolinite). Iron hydroxides were transformed to well-defined hematite (*d*_hkl_ 2.70 and 2.51 Å) and the feldspars remained intact.

**Figure 2:**
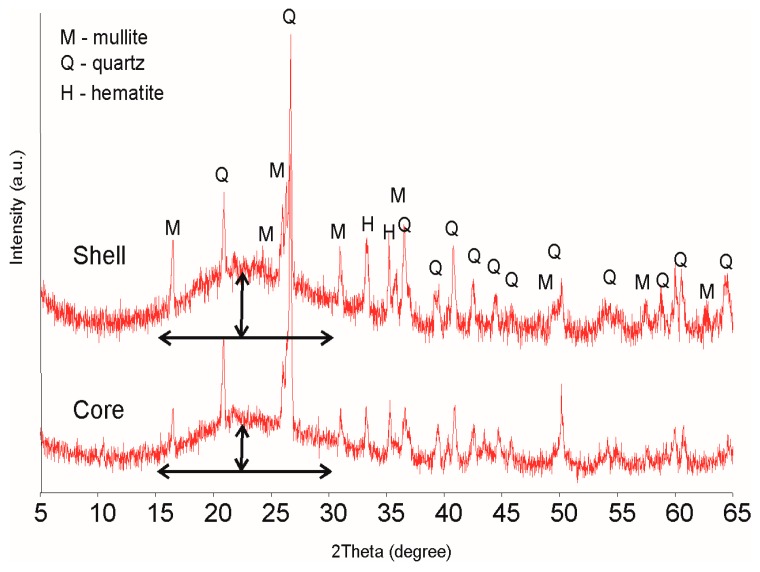
XRD patterns of internal (core) and external (shell) zones of lightweight aggregates (LWAs) (S + NAP 8%). Arrows show the height of the spectra from the basic line within a 2θ range of 15°–30°.

Apart from the above defined mineral phases, a significant contribution of the amorphous glassy phase could be distinguished by the rise of the spectra from the background line within a 2θ range of 15°–30°, as compared to the initial substrates spectra (not presented). Most of this glassy phase is located in the external vitreous layer (shell) of the obtained aggregates, as is evidenced in Figure 2 showing the XRD spectra of the shell and of the internal (core) materials of an exemplary LWA.

This glassy phase form well developed a vitrified layer on the external surfaces of all aggregates. However, Gonzales-Corrochano et al. [4] do not observe formation of such a layer in LWAs manufactured with used motor oil.

**Figure 3:**
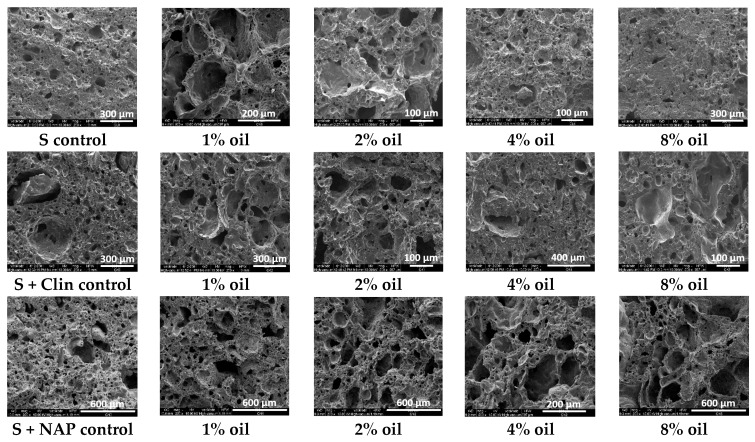
Representative scanning electron microscope (SEM) microphotographs of the studied aggregate sections.

SEM microphotographs of the obtained lightweight aggregates are presented in Figure 3. The presence of pores in fired aggregates is due to the thermal swelling of clay at high temperatures where the mineral material reaches the pyroplastic state and gases liberating from the raw material have sufficient vapor pressure to increase the pore volume [23]. Lightweight aggregates prepared from pure clay (S control) are characterized by a very compact texture and have the smallest pores. The addition of 1% oil leads to a sharp increase in the number of large pores which are surrounded by porous walls. A similar texture is observed for the addition of 2% oil whereas the addition of 4% oil seems to produce a more compact texture. After an 8% oil addition, the LWA texture returns to a very compact state, similar to the control aggregate. Control aggregates containing both zeolites pose some pores of much larger sizes than these occurring in the control clay aggregates (S). With the increase in oil content, the texture of the aggregates containing clinoptilolite behaves similarly to the clay aggregates. The LWAs containing NAP-1 have a highly porous texture, even at 8% oil addition.

**Figure 4:**
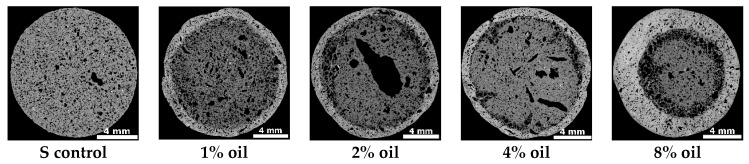

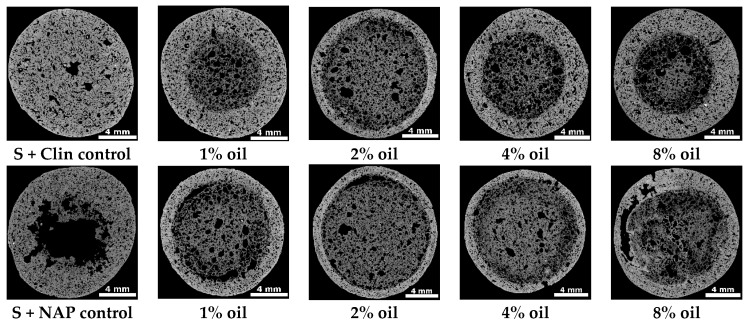
Exemplary 2D cross-section images derived from microtomography for the studied materials. Black areas are pores, white areas are solid.

Exemplary MT cross sections of the studied LECA’s are presented in Figure 4. The visual analysis of the scans reveals that the lightweight aggregates pose thick, dense areas, extending throughout the whole S and S + Clin and being very thick for S + NAP control aggregates. The thickness of these dense areas becomes small (around 12% of the aggregate radius, AR) for S aggregates containing 1%, 2% and 4% oil and rises again at 8% oil addition (around 40% of AR). For S + Clin aggregates, the thickness of the dense area extends to around half of the AR at 1% oil addition, reaches the minimum at 2% oil addition (around 12% of AR) and then rises again. A similar trend occurs for S + NAP aggregates, however the thinnest layer is produced for 2% and 4% oil addition. Generally, the dense area is the thinnest for all LWAs produced from substrates containing 2% oil.

**Figure 5:**
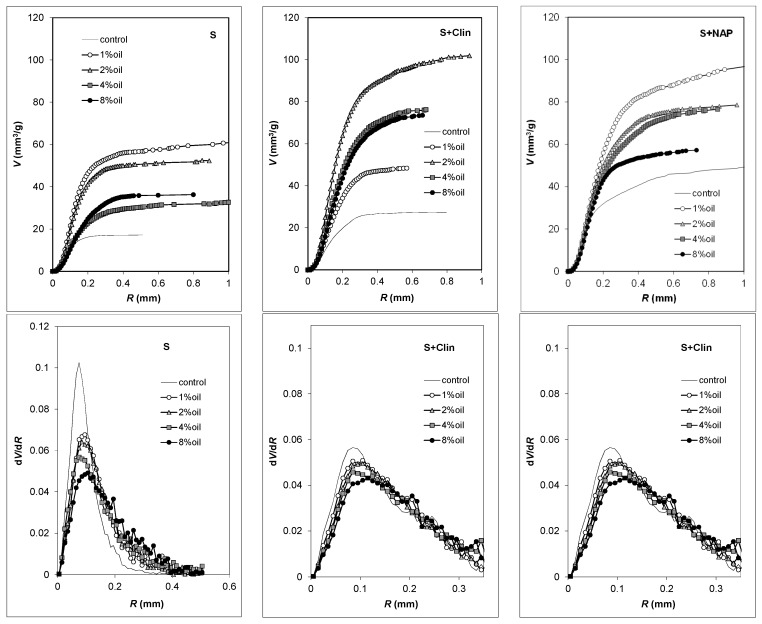
Pore volume vs. pore radius dependencies (**up**) and normalized pore size distribution functions (**down**) derived from microtomography scans. Average results from the experimental replicates are plotted.

Calculated from 3D MT scans, pore volume vs. pore radius dependencies and pore size distribution functions are presented in Figure 5. The control aggregates containing Na-P1 develop the largest pore volumes. In general, the pore volumes of all aggregates containing the motor oil are significantly larger than those of the control ones. The highest pore volume is developed at a 1% oil addition for clay and clay + NAP-1 aggregates, whereas for clay + clinoptilolite aggregates the highest pore volume is reached at 2% oil addition. For all LWAs, the pore size distribution functions are unimodal with the maxima located at around 0.1 mm pore radius. The sharpest peaks are observed for control aggregates and the oil addition leads to the peaks broadening.

**Figure 6:**
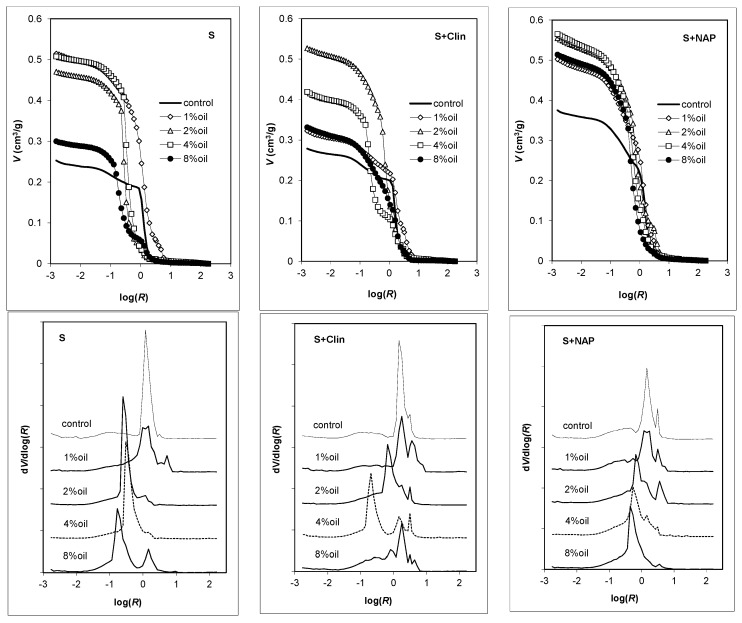
Mercury intrusion porosimetry curves (**up**) and normalized pore size distribution functions (**down**) for the studied aggregates. The average results for the experimental replicates are plotted. The unit of *R* is μm.

Mercury intrusion porosimetry curves, relating the intruded mercury (pore) volume to the logarithm of pore radius and the normalized pore size distribution functions for the studied materials, are presented in Figure 6. It is worth noting that the mercury extrusion branches were, in all cases, practically parallel to the log(*R*)-axis, indicating that practically all the mercury is accumulated in the pore voids and that the amount of the necks (channels) connecting these voids is negligible.

Similarly, as for MT pore volumes, the volume of intruded mercury to the control aggregates is the lowest for the LWA containing only the original clay deposit, medium for the LWA enriched with clinoptilolite and the highest for material containing Na-P1. For clay aggregates, the highest pore volume is developed at 1% oil, for clay + clinoptilolite aggregates at 2% oil and for clay + NAP-1 aggregates at 4% oil addition.

The pore size distributions of the control aggregates are less complicated (smaller number and sharper peaks) than for LWAs made from oil added substrates. The presence of oil shifts the dominant peaks towards smaller radii and some additional peaks are developed. Comparing the pore size distribution functions derived from MIP and MT, one can see that MIP measurements allocate the sizes of almost all the pore volumes towards an underestimation of the large pores and an overestimation of the small pores. This phenomenon, as summarized by Korat et al. [8], appears to be rather intrinsic than accidental, and it derives from the lack of direct accessibility of most of the pore volumes (including air voids) to the mercury surrounding the specimen. Furthermore, in the case of highly porous structures, errors can also be made due to the breaking of the inner pore’s walls, which then gives distorted results.

The MIP pores for all of the studied aggregates exhibited three linear fractality ranges: 40–1 μm, 0.7–0.1 μm and 0.02–0.001 μm, thus showing multifractal behavior, which is frequent in natural objects [39]. However, the slopes of the linear log–log plots were very high, so the calculated fractal dimensions of the pore surfaces were larger than 3 in practically all cases. Since the fractal dimensions for porous solids may vary from 2 to 3 [39] with the lower limiting value of 2 corresponding to a perfectly regular pore surface and the upper limiting value of 3 relating to the maximum allowed pore surface complexity, our results have no physical meaning. Those high “fractal dimensions” may result from the specific structure of the aggregates: if the large pores are accessible through markedly narrower entrances, the large pore volume is attributed to the radius of the entrance and because all the volume is treated as belonging to a long capillary in a cylindrical pore model, the d*V*/d*R* is also higher and gives *D* values higher than 3.

The pore parameters of the pore system of the control aggregates (without oil addition), derived from the experiments described above, are summarized in Table 1.

The total pore volumes and porosities measured by all methods are the highest for S + NAP and the lowest for natural clay (S) aggregates. As a rule, MIP measures significantly higher pore volumes than MT. The measuring range of MT starts from ~6 µm upwards and for MIP it is from ~0.004 to ~14 µm and, at first glance, it seems impossible that an MIP registers larger porosities. However, mercury can invade the whole aggregate interior through narrow entrances to the large pores and thus fill all large pores inside. Therefore, we rely more on the total porosity values measured from MIP then on these measured by MT.

LWAs made from the clay deposit have the smallest porosity and the highest bulk density, whereas amendment of the artificial Na-P1 zeolite induces the formation of the largest porosity and smallest bulk density (around 1.2 g/cm^3^). It is worth noting that very similar bulk densities of the aggregates are measured by MIP, MT and directly from the LWA volume and mass, which indicates that the amount of very fine pores being unavailable for mercury is very small in all LWAs obtained by us. The solid phase (skeletal) density is the highest for S + Clin and the smallest for S + NAP aggregates.

**Figure 7:**
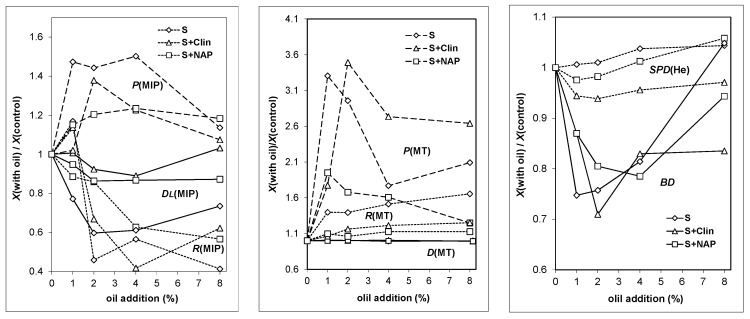
Relative changes in structural parameters of the LWAs with increasing oil content.

Relative changes in structural parameters of the LWAs with increasing oil content in the substrates are depicted in Figure 7. In this figure the *y*-axis shows the ratio of the given parameter for the aggregate produced with a given oil addition to the same parameter for the control aggregate.

The aggregates’ porosities, measured by MIP and MT, increase at smaller oil loads and then decrease at the highest load. The increase in porosities measured by MIP is markedly lower (up to 1.5 times) than for larger pores measured by MT (up to 3.5 times). At smaller loads, organic substances present in the oil produce additional gases during the sintering process, that contribute to the formation of pore beads and the creation of a more porous structure of the aggregate, as was postulated by Wang et al. [41]. At higher oil loads, this process may be too intensive and a smaller part of the released gases may be entrapped by the melted solid.

The relative changes in porosities are the lowest for S + NAP aggregates, most probably because they initially have the largest pore volumes and the input of expanding gas produced from oil is the smallest in large pores. The average pore radii, measured by MIP, tends to decrease with the increase of oil load, whereas these measured by MT increase slightly which may indicate different pore behavior in different ranges of their sizes. The smaller pores become smoother with the oil load increase, as indicated by the decrease in MIP “fractal dimensions”, whereas the build-up of larger pores remains unaltered as it can be concluded from constant values of MT fractal dimensions. All fractal dimensions calculated from microtomography data fall within the range between 2 to 3 and are rather high, indicating the complex pore buildup. The MT seems to provide a more realistic picture of the LWAs’ fractal pore structure than MIP which may be due to either the application of the other pore model (spherical instead of cylindrical pore spaces) or, more probably, to a failure of MIP application for the description of LWAs’ pore size distribution due to attributing the radii of pore entrances (necks) to the sum of the volumes of pore necks and voids. The bulk density of the aggregates, as could be expected, behaves inversely to the aggregates’ porosity. Solid phase density for aggregates containing pure clay (S) increases with an increasing oil load. After the initial drop at 1% oil, the SPD of S + Clin and S + NAP aggregates increases, also with higher oil loads. We suspected that the solid phase density should decrease due to the presence of residual coal in the aggregates, however, no coal was detected in any aggregate (grinded in a colloidal mill). The values of solid phase density, being lower than those for the control aggregates, may be caused by the effect of oil on the production of very small closed pores that are unavailable for He atoms. The occurrence of higher solid phase densities are, for us, not clear.

## 4. Conclusions

The lightweight aggregates prepared from beidellitic clay (containing 10% of natural clinoptilolite or Na-P1 zeolite), admixed with various doses of a used motor oil, were studied. The mineral composition of the aggregates was determined by X-ray diffraction and their microstructure was determined by a combination of mercury porosimetry (MIP), microtomography (MT), nitrogen adsorption/desorption isotherm (NA) and scanning electron microscopy (SEM). The pore structure of the LWAs prepared from a clay deposit was strongly modified by the addition of natural clinoptilolite and synthetic Na-P1 zeolite. The addition of used motor oil to the substrates used for LWAs’ production (clay and its mixtures with 10% of zeolites) markedly altered the aggregates’ pore characteristics, depending on the oil load. The porosity of the LWAs depended nonlinearly on the oil addition: maximum porosity measured by MIP and MT was produced at low (1%–2%) oil concentrations and it dropped at higher concentrations, opposite to the aggregates’ bulk density. The increase of porosities and pore volumes in a range of large pores (measured by microtomography) was markedly higher (up to 3.5 times) than this measured by mercury intrusion (up to 1.5 times). The presence of Na-P1 resulted in the highest porosity of the obtained aggregates. The most pronounced changes in the aggregates’ pore size distributions were observed by mercury intrusion porosimetry. Extremely small surface areas of LWAs were measured by NA. The mineral composition of the produced LWAs seemed to depend neither on the oil addition nor on the zeolitic admixtures and it was similar for all aggregates.

Admixing the substrates with different amounts of used motor oil allows the regulation of the porous structure of the produced lightweight aggregates in a broad range of applications, which enables the obtainment of materials dedicated to defined specific applications in various industrial purposes.

Since zeolitic materials are perfect sorbents of the motor oil, the used zeolitic sorbents obtained from e.g., the cleaning of roads after car accidents, appear to be valuable materials for lightweight aggregates’ production, which is an ecologically correct way to reuse the spent sorbents.

Large porosity creates interesting properties, such as low weight and good thermal and acoustic insulation, therefore, the addition of used motor oil, along with its sorbents, seems to be a very promising method to regulate the porous structure of lightweight aggregates. Such aggregates could be applied for backfilling behind retaining walls and bulkheads, pipe covering, footing and sub-basing for roads and parking lots, slope stabilizing, gas ventilation in landfills, and/or drainage; however, due to high porosity and low bulk density, their most feasible application may concern concrete admixing for reducing loads, and acoustic, and thermal insulation.

## Figures and Tables

**Table 1:** The structural parameters of the control lightweight aggregates (LWAs).

Structural Parameter	S	S + Clin	S + NAP
Solid phase density from helium pycnometry, SPD(He), g/cm^3^	2.20	2.29	2.19
Bulk density from aggregate mass and volume, BD, g/cm^3^	1.39	1.37	1.21
Data from mercury intrusion porosimetry (MIP)			
Total pore volume, *V*(MIP), cm^3^/g	0.25	0.28	0.38
Average pore radius, *R*(MIP), μm	5.9	7.7	6.5
Bulk density, BD(MIP), g/cm^3^	1.44	1.39	1.22
Solid phase density, SPD(MIP), g/cm^3^	2.26	2.27	2.23
Porosity, *P*(MIP), %	36.3	38.7	45.6
Fractal dimension, *D*_L_, for 40–1 μm large (L) pores	6.31	4.86	5.09
Fractal dimension, *D*_M_, for 0.7–0.1 μm medium (M) pores	3.50	3.23	2.71
Fractal dimension, *D*_S_, for 0.02–0.001 μm small (S) pores	3.71	3.51	3.47
Data from microtomography (MT)			
* Pore volume, *V*(MT), mm^3^/g	17.4	27.6	44.2
Porosity, P(MT), %, including all pores	15.5	19.6	27.4
* Average pore radius, *R*(MT), μm	10	15	16
Fractal dimension, *D*(MT)	2.75	2.76	2.76
Bulk density, BD(MT), g/cm^3^	1.39	1.39	1.17

* These two parameters were calculated in the same pore range, 0–510 μm, that was common for all aggregates. This allowed for better comparison of the results by exclusion of large pores, present more or less accidentally in different samples.
